# Salmon increase forest bird abundance and diversity

**DOI:** 10.1371/journal.pone.0210031

**Published:** 2019-02-06

**Authors:** Marlene A. Wagner, John D. Reynolds

**Affiliations:** 1 Earth to Ocean Research Group, Department of Biological Sciences, Simon Fraser University, Burnaby, British Columbia, Canada; 2 Hakai Institute, Heriot Bay, British Columbia, Canada; University of South Carolina, UNITED STATES

## Abstract

Resource subsidies across ecosystems can have strong and unforeseen ecological impacts. Marine-derived nutrients from Pacific salmon *(Onchorhycus spp*.*)* can be transferred to streams and riparian forests through diverse food web pathways, fertilizing forests and increasing invertebrate abundance, which may in turn affect breeding birds. We quantified the influence of salmon on the abundance and composition of songbird communities across a wide range of salmon-spawning biomass on 14 streams along a remote coastal region of British Columbia, Canada. Point-count data spanning two years were combined with salmon biomass and 13 environmental covariates in riparian forests to test for correlates with bird abundance, foraging guilds, individual species, and avian diversity. We show that bird abundance and diversity increase with salmon biomass and that watershed size and forest composition are less important predictors. This work provides new evidence for the importance of salmon to terrestrial ecosystems and information that can inform ecosystem-based management.

## Introduction

Resource availability and movement are major processes shaping ecosystem structure and function [1]. Resource subsidies are prevalent across landscapes [2,3], and can have profound direct and indirect impacts on recipient community structure, influencing primary productivity, trophic interactions, and predator-prey relationships [1,4–6]. Coastal streams provide bidirectional highways for nutrient transport [7], and support the anadromous and semelparous life-history of Pacific salmon (*Oncorhynchus spp*.). Salmon deliver an annual and predictable flux of nutrient subsidies from marine to terrestrial systems, creating an opportune natural experiment to examine effects of variation in resource subsidies across ecosystem boundaries.

Pacific salmon acquire 99% of their body mass after leaving freshwater streams to grow and mature at sea [8]. When they return to spawn in natal streams, they bring a seasonal influx of marine-derived nutrients that enhance both freshwater and terrestrial productivity by fertilizing otherwise nutrient-poor watersheds with nitrogen and phosphorous [9–12]. Salmon carcasses are transferred to adjacent terrestrial habitat by bears, wolves, and other primary consumers, as well as through flooding and hyporheic flow [13–15].

After transfer, marine-derived nutrients move through several trophic pathways. First, they enhance primary production, favoring plant growth and structural complexity [5,16,17] and they influence the diversity of understory vegetation [12,18]. Birds respond to plant structure and composition, and discriminate habitat at fine scales associated with foliage density and geometry [19–21]. Furthermore, herbivorous insects are attracted to foliage with elevated levels of nitrogen [22–24], and both terrestrial and aquatic invertebrates can be more abundant on salmon streams [10,25,26] (but see [27,28]). As the combination of habitat (vegetation) and food (invertebrates) are essential resources for birds, salmon may play a role in shaping avian communities.

Songbird densities have been positively associated with artificial fertilization (N) of lakes [29]. Previous work also suggests that songbirds achieve higher densities in the presence of salmon across 15 small streams with and without salmon in Alaska [30], and this was supported in a comparison of bird densities above and below salmon barriers in two salmon-bearing and one stream without salmon at our study streams in British Columbia [31]. Additionally, both density and (Shannon’s) diversity of bird communities increased in estuaries in British Columbia along with salmon biomass [32,33]. However, no study has explored how salmon-derived nutrients affect birds in riparian forests across a range of salmon-spawning magnitudes. Our comparisons involved a wide span of salmon densities, as opposed to comparisons where salmon are either presence or absent, and we test for correlations with bird communities across as opposed to within watersheds [31]. We also standardize elevation and other factors that may influence bird communities inland by restricting site locations to forests near the mouths of streams.

## Materials and methods

### Study system

Our research was conducted under Simon Fraser University Animal Care Protocol #1044B-12, and approval from Heiltsuk Tribal Council. We conducted our study in Heiltsuk First Nation territory near Bella Bella, along the Central Coast of British Columbia, Canada (52.1619N, 128.1450W). This region is in the Western Hemlock Coastal Biogeoclimatic Zone, characterized by a cool, maritime climate, heavy rainfall (>3 m per year), and forests dominated by coniferous tree communities of western hemlock *(Tsuga heterophylla)*, and Sitka spruce *(Picea stichensis)*, western red cedar *(Thuja plicata)*, and Amabilis fir *(Abies amabilis)*. The only deciduous tree in the region is red alder *(Alnus rubra)*. Understory vegetation consists of stink currant *(Ribes bracteosum)*, blueberry and huckleberry *(Vaccinium spp*.*)*, salmonberry *(Rubus spectabilis)*, salal *(Gaultheria shallon)*, false azalea *(Menziesia ferruginea)*, red elderberry *(Sambucus racemosa)*, and devil’s club *(Oploplanax horridus)*. Further habitat descriptions are in Mathewson et al. 2003 and Hocking & Reynolds 2011.

**Fig 1:**
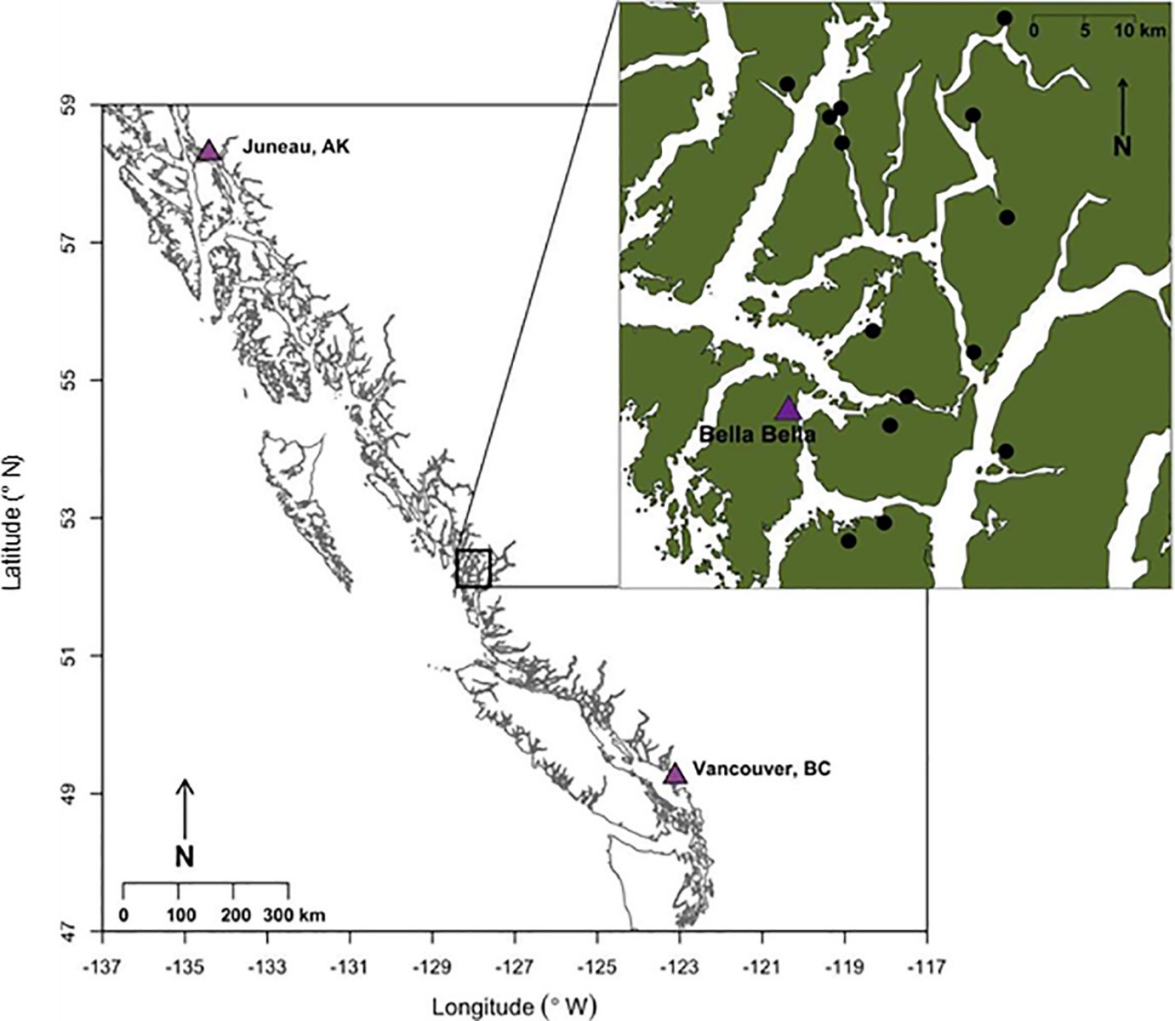
Location of 14 study streams along the central coast of British Columbia.

Study sites in 14 watersheds that span approximately 60 km of the coast were chosen because they were adjacent to streams supporting a wide range of salmon (0 to 122,454 fish; Fig 1). Selective logging occurred along some streams in the mid-twentieth century, however no streams had been clear-cut and other modern-day anthropogenic disturbance is slight or nonexistent. All sites were only accessible by boat from the sea.

### Breeding birds

We conducted standard 10-minute point-count surveys [34] over two years across the streams in 14 watersheds, where all birds seen or heard were recorded. Point-count stations were positioned in the riparian forest on alternate sides of the stream, beginning 50 m inland from the estuary. Each point count was located 50 m upland from the stream, for a total of 5 point-counts on each stream, located 100 m apart. Each station was surveyed 4 times between May 16 and July 23, 2012, and 3 times between May 16 and July 17, 2013. All surveys were completed between 6:30 and 10:20 am PST. Each stream was visited once in a rotation before continuing the process over again with starting points reversed on subsequent visits. Point-count surveys were not conducted in heavy rain or wind (>3; Beaufort Scale), or at stream noise levels rated more than moderate on a standard ordinal scale (0–5; [31]). If conditions prevented censusing, we returned during the same rotation under better conditions.

Birds detected as fly-overs, non-forest dwelling birds (e.g. seabirds, herons, gulls, etc.), and birds detected on fewer than two surveys (early spring migrants and other non-breeders) were excluded from analyses. We also excluded American Dipper *(Cinclus mexicanus)*, an obligate riverine songbird that will include salmon eggs and fry in their diet [35], because we were interested in the influence that salmon may have on birds through indirect pathways. We truncated the bird data to include only detections within a 100 m radius due to the small overall plot size (~10 ha), distance between point-counts, and to ensure that resources within the riparian zone were available to our sample population. If birds were detected at more than one count, we removed subsequent detections from analyses. We calculated total bird density as the relative abundance at each point-count for all bird species combined [36]. We then separated the analyses into generalist, insectivore, and frugivore foraging guilds, and we examined the six most commonly detected species across all study sites for each year. Finally, because local bird species richness and available energy resources should be positively correlated, we calculated two diversity indices, the effective number of species [37], and species richness (the number of species observed at each point count station). The effective number of species represents the number of equally common species, is the exponential of Shannon’s Diversity, and has the advantage of scaling linearly with species richness [38–40]. Subsequently, bird response variables were grouped into three general categories: 1) relative abundance of all birds and foraging guilds, 2) relative abundance of individual bird species, and 3) avian diversity measures.

### Salmon

Collaboration between the Department of Fisheries and Oceans, the Heiltsuk Integrated Resource Management Department, and our research group at Simon Fraser University has resulted in the creation of a large salmon dataset within the region, with salmon counts conducted annually each fall. On each stream, the number of pink *(O*. *gorbuscha)* and chum *(O*. *keta)* salmon, both live and dead, were counted by walking streams before, during, and after the peak spawning period. Other species of salmon were excluded from analyses because they comprise less than 5% of the salmon populations in these streams and tend to spawn farther upstream.

We used salmon count data from 2009 to 2011 to quantify a 3-year average salmon biomass metric (Table 1). On streams where 3 counts had been repeated, the area-under-the-curve (AUC) method was used to estimate the total number of spawning fish for the year [41]. Otherwise, the peak counts of live + dead were used as totals, which result in very similar population estimates to the AUC method [12]. Biomass was estimated using:

$$Salmon\;biomass=\Sigma \;(N_{i}\times W_{i})$$

where *N* = the number of adult salmon, *i* = the salmon species; pink or chum, and *W* = regional salmon mass estimates (1.2 and 3.5 kg, respectively; [12]). We also calculated two alternative metrics of salmon density:

$$Salmon\;density/m^{2}=\Sigma \;\left(N_{i}\times W_{i}\right)/L\times W$$

and

$$Salmon\;density/m=\Sigma \;\left(N_{i}\times W_{i}\right)/L$$

where *L* = the spawning length of the stream and *W* = the mean bankfull width (the maximum width of a stream channel before flooding) of the stream, as these are common measures of salmon indices found in the literature. Preliminary analysis using univariate linear models with each salmon metric indicated that they were highly correlated and that our salmon biomass estimates (rather than density) explained the most variation in our bird data, so we used this measurement in all subsequent analyses (S1 Table).

**Table 1:** Stream-specific features. **Watershed, stream, and salmon metrics across 14 streams the central coast of British Columbia.** Salmon biomass was calculated from 2009–11 mean counts of spawning adults.

Stream	Watershed catchment area (km^2^)	Bankfull width (m)	Spawn length (m)	Salmon biomass (kg)
Beales	6.5	10.9	300	2,544
Bullock	3.3	10.9	622	13,558
Clatse	24.3	22.8	900	48,040
Fancy	9.9	4.8	298	922
Fannie	16.4	12.8	1,500	26,200
Farm Bay	2.3	6.4	0	0
Fell	7.0	10.9	0	0
Goatbushu	4.5	7.5	550	2,193
Hooknose	14.8	16.9	1,800	12,475
Kill	0.5	3.5	453	5,277
Kunsoot	4.9	13.1	1,280	1,242
Neekas	16	17.7	2,100	154,402
Quartcha	29.4	21.7	5,500	14,447
Ripley	15.4	14.7	0	0

### Forest habitat

Forest habitat variables were quantified from data collected on 50 m vegetation transects running perpendicular from the stream to point-count stations. Diameter at breast height (DBH), and percent cover by species and height class (1.3–15 m, 15–25 m, and >25 m) were recorded for all trees greater than 2.5 cm DBH in a 6 m belt along each transect. Shrub cover was recorded as the percentage of cover by species and height class (<0.5 m, 0.5–1.3, and >1.3 m; including saplings with DBH <2.5 cm) in five 1 m^2^ quadrats located at 5, 15, 25, 35, 45 m along the transects. Vegetation sampling methods were adopted from modified protocols described in Christie and Reimchen (2008) and Field and Reynolds (2011).

The DBH data were used to calculate stand basal area per plot for each dominant conifer tree species and subjected to a principal components analysis (PCA) to determine the major axes of change in conifer composition. Mean percent cover estimates from the six height classes for each transect were used to determine foliage height diversity (FHD; [42]). We also calculated the mean percent cover of red alder, a nitrogen-fixing species and the only deciduous tree in the region, and the mean percent cover of shrubs per transect (Table 2).

**Table 2:** Forest composition features. **Mean stand basal area for conifer species, mean percent cover for red alder and shrubs, and foliage height diversity (FHD) across 14 streams along the central coast of British Columbia.** The FHD represents the distribution in amount of vertical canopy within plots.

Stream	Amabilis fir	Sitka spruce	Snags	Western red cedar	Western hemlock	% cover red alder	FHD
Beales	10.5	43.4	3.2	0.1	11.1	14.2	1.3
Bullock	9.9	7.2	45.5	0	36.7	43	1.6
Clatse	0	20.2	80.0	0	25.2	44.8	1.3
Fancy	6.3	0.1	21.3	87.4	15.9	0.6	1.7
Fannie	18.9	8.8	23.4	52.8	16.1	14.5	1.4
Farm Bay	14.7	6.2	21.8	59.1	25.3	14	1.7
Fell	4.1	6.2	24.9	83.7	13.8	27	1.7
Goatbushu	5.9	13.2	16.9	16.6	29.2	39.5	1.5
Hooknose	8.1	50.9	56.4	0.2	22.6	5	1.6
Kill	3.2	1.0	13.7	26.1	31.7	17	1.6
Kunsoot	10.2	11.3	27.7	6.9	23.5	0	1.6
Neekas	0.6	16.5	5.4	0	18.4	35	1.5
Quartcha	11.3	53.5	19.1	4.9	13.2	19	1.6
Ripley	3.9	24.4	47.2	58.2	21.5	7.5	1.7

Principal components of conifer composition PC1 and PC2 explained 23.4% and 21.2%, respectively, of the cumulative variation in forest species across 70 vegetation plots (S2 Table). PC1 for conifer composition indicates a shift from spruce-dominated to cedar-dominated riparian areas, with negative loadings for stand basal area of Sitka spruce (-0.65) and Amabilis fir (-0.38), and high stand basal area of western red cedar (0.64). PC2 represents a shift from low Amabilis fir (-0.45) to high Western hemlock (0.61) and snags (0.63).

### Watershed size

Variation in geomorphology shapes riparian structure within a watershed [43]. Watershed size can influence cross-boundary nutrient transfer by mediating both predator access to salmon and terrestrial inputs [44,45]. Watershed catchment area (km^2^) was calculated in a Geographical Information System using iMAPBC ([46], Table 1). To account for stream size and length, we used field measurements of bankfull width and the mainstream salmon spawning length [12]. We combined these three variables to compute a PCA for overall watershed size for each of our study streams. The first principal component axis explained 82% of the overall variation in catchment area, bankfull width, and length of stream used by salmon, with all variables loading positively (S2 Table). This was therefore the only metric used in subsequent analyses to describe watershed characteristics.

### Analyses

To test hypotheses about the influence of salmon-spawning biomass on birds, we used hierarchical partitioning and Akaike’s Information Criterion adjusted for small sample sizes (AICc) with mixed-effects models. First, we modeled the individual contribution of each of our five forest habitat covariates (conifer composition PC1, conifer composition PC2, FHD, red alder cover, and shrub cover), on each of our bird response variables. The top-ranked forest habitat variable was then used to build candidate model sets. We used this method to limit insertion of forest habitat covariates in final candidate model sets [47], as our intent was to test overall influences of factors that may drive bird distribution and diversity in the watershed, and not quantify fine-scale habitat selection by various guilds or species.

We created a final candidate suite of seven models for each bird response variable, using the unique forest habitat covariate retained in our initial model selection process (S3 Table). We fit models to describe bird communities as a function of salmon biomass, forest habitat, and watershed size with site and point as random factors in all models to account for the repeated measures and spatial autocorrelation inherent in our study design [48]. We tested for multicollinearity among predictor variables. All variance inflation scores were less than two, and correlation coefficients were below 0.6. Yearly variation was included in all models as a two-level factor (2012 and 2013). We examined the variance structure of residuals to ensure assumptions of normality were met. Models were averaged to obtain weighted parameter estimates for highly competitive models (ΔAICc < 2) using the natural method [49]. We standardized individual coefficients to enable direct comparison of effect sizes across variables [50]. All analyses were completed in the R statistical program, version 3.2.2 [51], using packages *AICcmodavg* [52], *Mumln* [53], *nlme* [54], and *vegan* [55].

## Results

We detected 55 species of birds across our point-count surveys over both years, of which 35 were retained for final analyses (S4 Table). The six most commonly detected species were, in order of decreasing abundance; Pacific-slope Flycatcher *(Empidonax difficilus)*, Pacific Wren *(Troglodytes pacificus)*, Golden-crowned Kinglet *(Regulas satrapa)*, Townsend’s Warbler *(Dendroica townsendi)*, Varied Thrush *(Ixoreus naevius)*, and Swainson’s Thrush *(Catharus usulatus)*.

**Fig 2:**
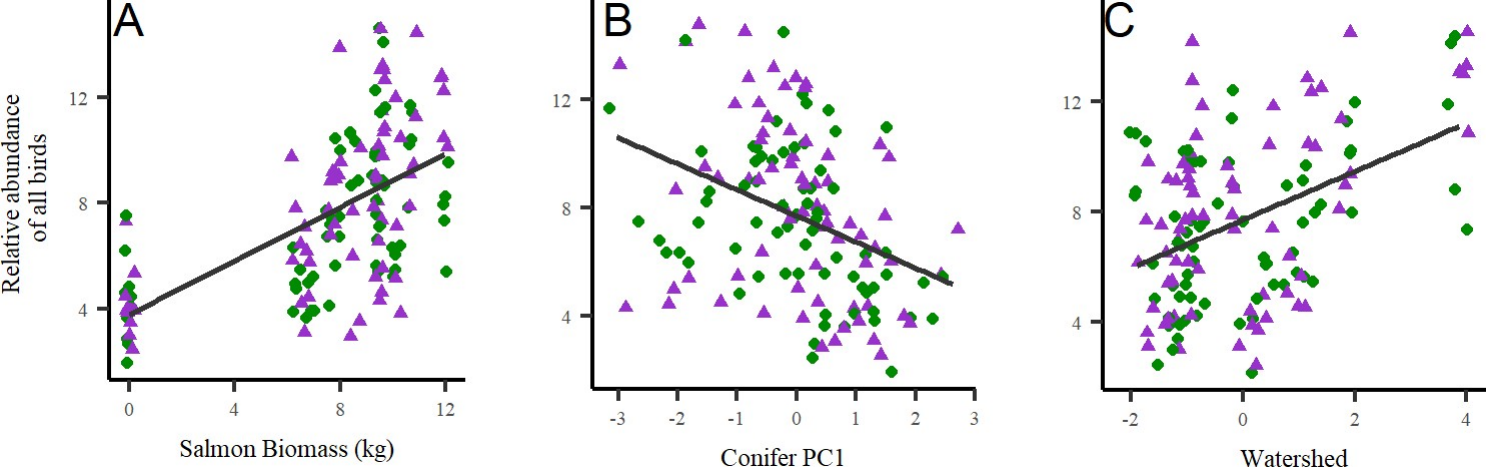
**Relationships between relative abundance of all birds and A) salmon biomass, B) conifer composition, and C) watershed size.** Salmon biomass was log-transformed and 2012 (blue triangles) and 2013 (green circles) data points were jittered to prevent over-plotting. Conifer composition and watershed size metrics are based on PCA (see methods; S2 Table).

**Fig 3:**
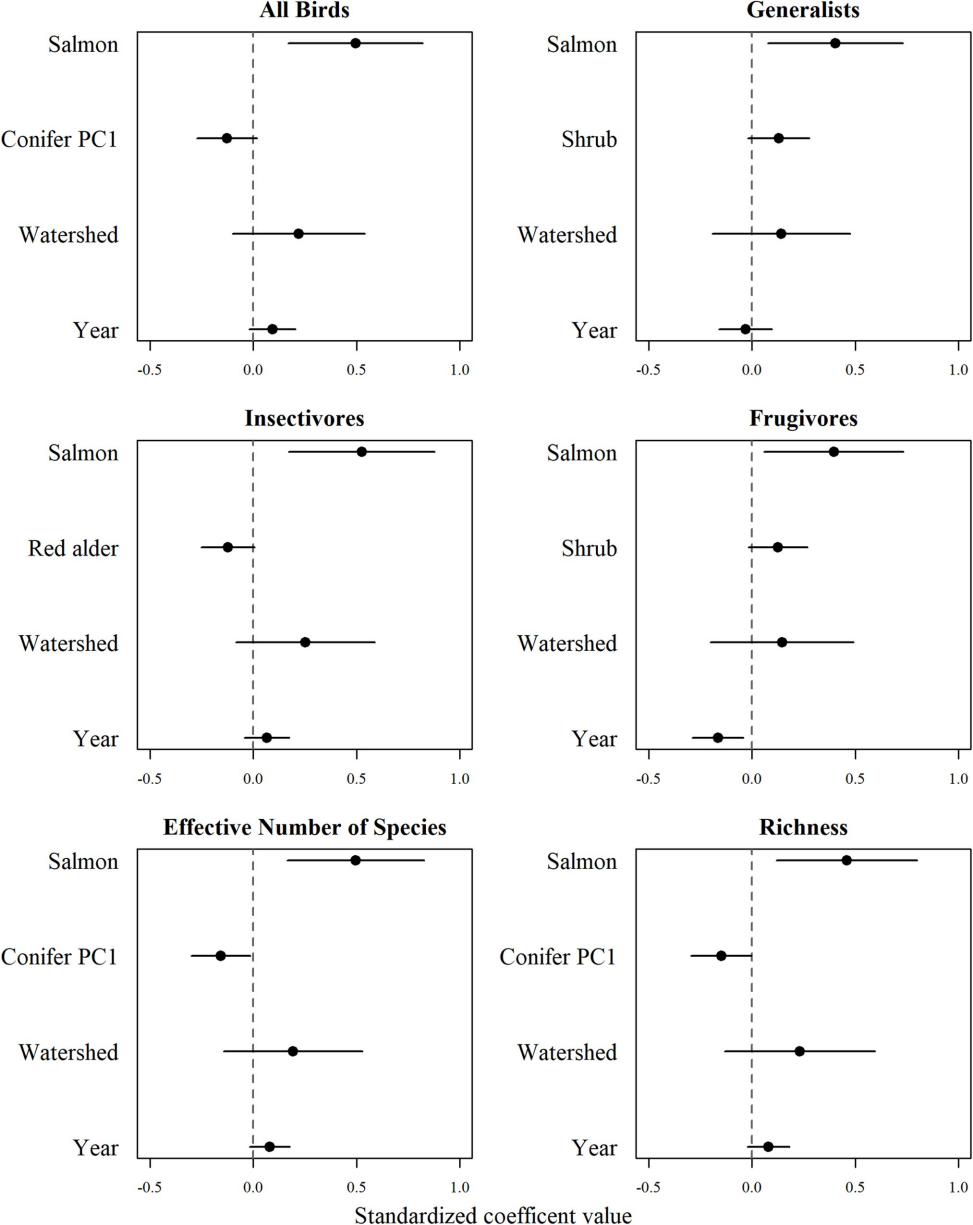
Standardized coefficients with 95% confidence intervals from the averaged mixed effects model (ΔAICc < 2) for all birds, generalists, insectivores, frugivores and diversity metrics of effective number of species and richness as a function of salmon, forest habitat, and watershed size.

**Fig 4:**
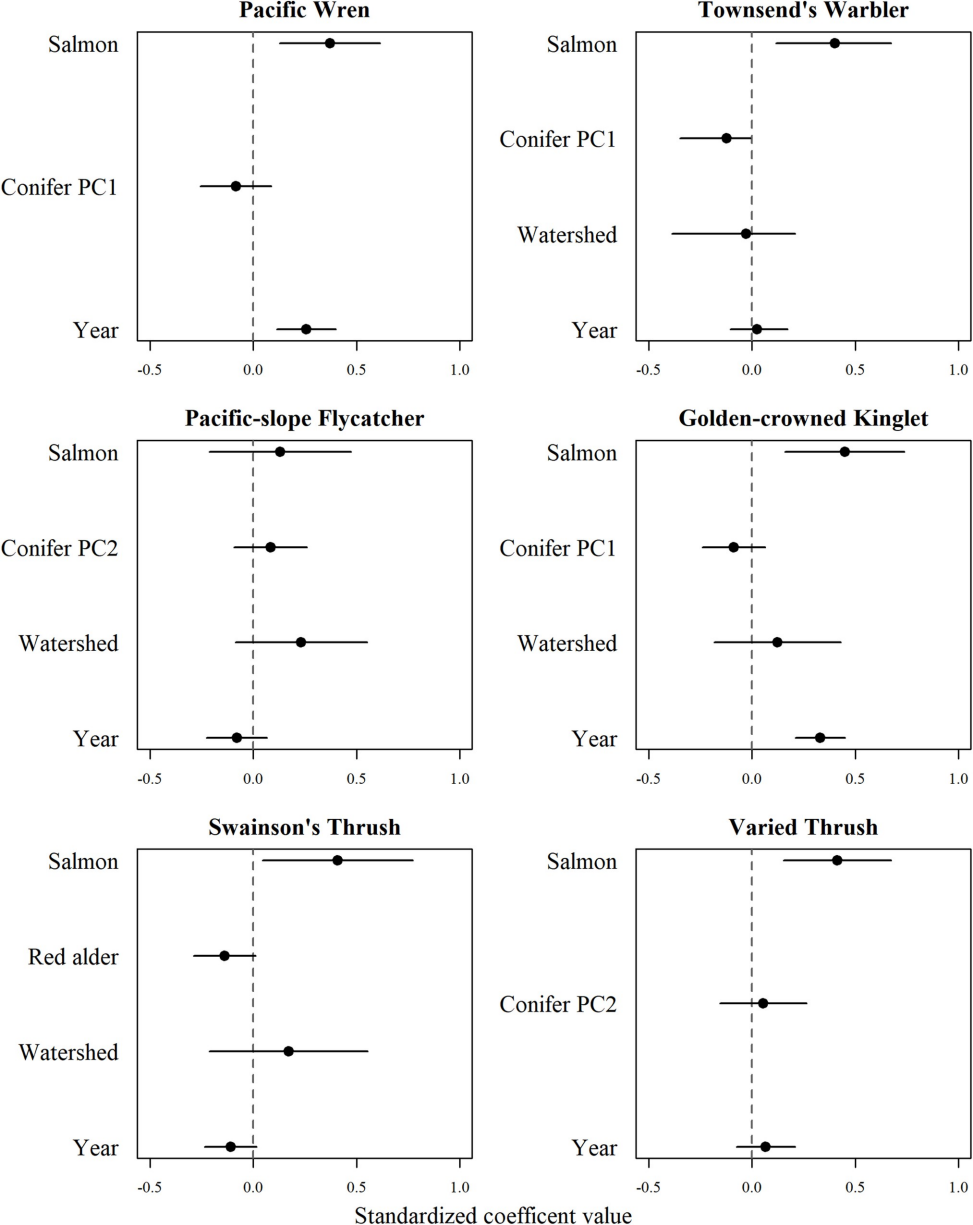
Standardized coefficients with 95% confidence intervals from the averaged mixed effects model (ΔAICc < 2) for the 6 most common species as a function of salmon, forest habitat, and watershed size.

Streams with higher salmon biomass had a greater relative abundance of all birds detected, and this was true within each of our foraging guilds of generalists, insectivores, and frugivores (Figs 2 and 3). We also observed a positive relationship between salmon biomass and effective number of species and species richness (Fig 3). Each individual species, except for Pacific-slope Flycatcher and Varied Thrush, showed strong evidence of higher relative abundances as salmon biomass increased (Fig 4).

Most top-ranked models in our final analysis retained a forest habitat covariate (Table 3). Abundances for all birds combined, Pacific Wren, Townsend’s Warbler, Golden-crowned Kinglet, and both diversity measures increased with salmon biomass and decreased conifer PC1, indicating a preference for stands that are fir- and spruce-dominated (Figs 2–4). Generalist and frugivore abundances also increased with salmon biomass and with shrub cover (Fig 3). Insectivore and Swainson’s Thrush abundances increased with salmon biomass but decreased with red alder (Figs 3 and 4; S5 Table).

**Table 3:** Model selection results (ΔAICc < 2) depicting avian response to stream, salmon, and habitat features on 14 streams along the central coast of British Columbia. Year was included as a covariate in all models but is not included in the table for clarity.

Avian Response	Model	*k*	*logLik*	ΔAICc	*w*
All birds	Salmon, Conifer PC1	7	-304.6	0	0.31
	Salmon, Watershed, Conifer PC1	8	-303.6	0.2	0.28
	Salmon, Watershed	7	-305	0.8	0.21
	Salmon	6	-306.4	1.3	0.16
Generalists	Salmon, Shrub	7	-208.9	0	0.35
	Salmon	6	-210.5	1	0.21
	Salmon, Shrub, Watershed	8	-208.4	1.3	0.19
Insectivores	Salmon, Watershed, Red Alder	8	-290.1	0	0.34
	Salmon, Red Alder	7	-291.4	0.3	0.29
	Salmon, Watershed	7	-291.8	1.1	0.2
	Salmon	6	-293.3	1.8	0.14
Frugivores	Salmon, Shrub	7	-201	0	0.35
	Salmon	6	-202.7	1.1	0.21
	Salmon, Watershed, Shrub	8	-200.6	1.3	0.18
Effective number of species	Salmon, Conifer PC1	7	-192.4	0	0.39
	Salmon, Watershed, Conifer PC1	8	-191.6	0.6	0.29
Richness	Salmon, Conifer PC1	7	-211.4	0	0.36
	Salmon, Watershed, Conifer PC1	8	-210.4	0.3	0.32
Pacific Wren	Salmon	6	-136.6	0	0.47
	Salmon, Conifer PC1	7	-136.1	1.2	0.25
Townsend's Warbler	Salmon	6	-98	0	0.39
	Salmon, Conifer PC1	7	-97	0.3	0.34
Golden-crowned Kinglet	Salmon	6	-87	0	0.4
	Salmon, Conifer PC1	7	-86.4	1	0.25
	Salmon, Watershed	7	-86.5	1.3	0.21
Swainson's Thrush	Salmon, Red Alder	7	-74.1	0	0.33
	Salmon	6	-75.8	1.1	0.19
	Salmon, Watershed, Red Alder	8	-73.6	1.2	0.18
Varied Thrush	Salmon	6	-103.6	0	0.24
	Salmon, Conifer PC2	7	-102.7	0.3	0.21
	Salmon, Watershed	7	-103.1	1.1	0.14
	Salmon, Watershed, Conifer PC2	8	-102	1.3	0.12
	Watershed	6	-104.4	1.6	0.11
	Watershed, Conifer PC2	7	-103.3	1.6	0.11

Overall watershed size characteristics that included width and length of the streams, and catchment size, explained little of the variation in our bird metrics. However, overall watershed size was included in two highest-ranking models (insectivores and Pacific-slope Flycatcher; Figs 3 and 4; Table 3), and occurred across most averaged models (except Pacific Wren, Townsend’s Warbler). However, confidence intervals for effect sizes overlapped zero, leading to ambiguity in results.

## Discussion

Our study confirms that salmon may provide an important indirect resource subsidy for songbirds. We controlled for both watershed size and forest habitat while examining the influence of salmon on avian communities across 14 streams that supported a wide range of variation in biomass of spawning salmon. Many of the species in this study are migratory, and therefore they do not have direct exposure to salmon in the fall (S4 Table), but are responding to their influence in the spring. This provides evidence for a seasonal legacy effect of salmon. Every bird metric correlated positively with salmon biomass, with the exception of the relative abundance of Pacific-slope Flycatcher (which was not explained well by any model), and the relationship with salmon was greater than either forest habitat or watershed size. Our study corroborates with previous work on two of our study streams that found increased abundance of Pacific Wren, Swainson’s Thrush, and Golden-crowned Kinglet on salmon-bearing reaches compared to non-salmon bearing reaches above waterfalls [31]. Additionally, our results also provide strong evidence that inputs of salmon even at low levels on streams appear to elevate bird populations above those without salmon.

There are likely several pathways that provide birds with the benefits from salmon biomass [56]. Salmon enhance primary productivity in aquatic ecosystems [57], thus increasing the density of common invertebrate taxa, at least in the spring [10,26,58,59]. In the spring, emergent aquatic insects can comprise 50–90% of resident bird diet, at a time when other invertebrate populations are low [60,61]. Concentrations of both migrant and resident birds correlate strongly with the timing of emergence [62,63] and emergent population abundance [64]. Many terrestrial invertebrates oviposit directly on salmon carcasses [65]. For example, Calliphoridae exhibit high productivity when breeding on chum carcasses and contribute to a significant increase in overall invertebrate numbers in the fall [30,45]. Furthermore, herbivorous insects select vegetation with high levels of nitrogen and may attain higher densities on vegetation subsidized by salmon [22–24,66]. We also recorded higher numbers of terrestrial invertebrates at nests in forests along streams with more fish in the breeding season during field collections (Wagner, unpublished data).

Forest habitat influenced many of our avian response variables. Overall, conifer composition was the strongest forest habitat influence on abundance for all birds and for Pacific Wren, Townsend’s Warbler, and Golden-crowned Kinglet. These results indicate a preference for spruce- and fir-dominated forests and an avoidance of cedar. Tree species composition is important to insectivores [67,68], and members of the cedar family have secondary chemicals that reduce invertebrate colonization of their bark and branches. Cedars host fewer beetles compared with both Western hemlock and Amabilis fir, and this in addition to repellant phenolics may reduce the prevalence of bark-gleaning or other foraging birds [69,70]. Surprisingly red alder, which fixes atmospheric nitrogen and is the only deciduous tree present at study sites, was a negative predictor for both insectivores and Swainson’s Thrush, which was contrary to our expectations, though 95% confidence intervals overlapped zero in both analyses. Shrub cover was a positive predictor for both generalists and frugivores, and this may reflect an attraction to berry-producing shrubs such as salmonberry, which are more dense along streams with more salmon [12].

Food availability is a major driver of songbird productivity [71]. Limitation of food resources can mediate spatial aggregation, nest success, and alter life history strategies. A passerine that does feed on salmon and eggs when available, the American Dipper, is more likely to disperse away from breeding territories in the winter in habitats without salmon, and enjoy higher breeding success with salmon [72]. While they consume salmon fry and eggs, much of their diet consists of aquatic invertebrates [73,74], suggesting indirect salmon subsidies also influence habitat use by this species. There is also evidence of salmon-derived nutrients in the diet of Pacific Wrens in two of the streams that we studied [75]. Further research is needed to see what additional mechanisms lead to the increased abundance of the birds that do not consume salmon or their eggs. For example, they could have higher nesting success on streams with more salmon, or they may preferentially aggregate on such streams [76].

## Conclusions

Our study shows that salmon biomass has a stronger relationship with bird density and diversity across watersheds than forest composition or watershed size. As salmon impact terrestrial taxa even in severely degraded habitats [77], and recently restored habitats [74], our results emphasize the strength and importance of cross-boundary subsidies. The current fishery management paradigm of maintaining stock levels for next generation recruitment does not consider essential landscape-scale processes that support ecosystem function. Our results also reflect the importance of considering cross-boundary interactions during the current trend towards ecosystem-based management [56,78–80]. As salmon runs persist well below historic levels in many parts of their North American range [81], an ecosystem-based approach to managing salmon and river ecosystems is necessary to maintain holistic ecosystem function.

## Supporting information

S1 Table Preliminary univariate models comparing three salmon density metrics. (PDF)

S2 Table Principal component factor loadings for conifer composition and watershed size. (PDF)

S3 Table Model selection results of all models depicting avian response to salmon and habitat features on 14 streams along the Central Coast of British Columbia. (PDF)

S4 Table Species, their associated foraging guilds, and migratory status of birds detected on point-count surveys in 2012 and 2013 along the Central Coast of British Columbia. (PDF)

S5 Table Fixed-effect estimates (standardized regression coefficients) and standard errors for averaged candidate models (ΔAIC<2) describing relative bird abundance as a function of salmon biomass, forest habitat, and watershed size across 14 streams along the Central Coast of British Columbia. (PDF)
