# Supplementary material for: Salmon increase forest bird abundance and diversity
Source: PLoS One. 2019 Feb 6;14(2):e0210031. doi: 10.1371/journal.pone.0210031 (PMC6364887; doi:10.1371/journal.pone.0210031)
Supplement: S1 Table — (PDF) [file pone.0210031.s001.pdf]

**S1 Table.** Preliminary univariate models comparing three salmon density metrics.

| **Salmon Metric** | ***logLik*** | **AICc** | **ΔAICc** | ***w*** | ***r*** **²** |
|---|---|---|---|---|---|
| Salmon Biomass (kg) | -325.6 | 657.5 | 0 | 1 | 0.32 |
| Salmon Density ($m^{-1}$) | -331.5 | 669.2 | 11.7 | 0 | 0.25 |
| Salmon Density ($m^{-2}$) | -341.7 | 689.7 | 32.2 | 0 | 0.14 |
