# Supplementary material for: Salmon increase forest bird abundance and diversity
Source: PLoS One. 2019 Feb 6;14(2):e0210031. doi: 10.1371/journal.pone.0210031 (PMC6364887; doi:10.1371/journal.pone.0210031)
Supplement: S2 Table — (PDF) [file pone.0210031.s002.pdf]

S2 Table. Principal component factor loadings for conifer composition and watershed size.

Analysis	Covariate	PC1	PC2
Conifer composition	Amabilis fir	-0.377	-0.453
	Sitka spruce	-0.649	0.017
	Snag	-0.157	0.63
	Western red cedar	0.637	-0.174
	Western hemlock	0.082	0.606
	% explained	*23.4*	*21.1*
Watershed size	Catchment area (km^2)	0.606	-0.263
	Bankfull width (m)	0.588	-0.488
	Spawn length (m)	0.536	0.832
	% explained	*82.0*	*14.0*
