# Supplementary material for: Salmon increase forest bird abundance and diversity
Source: PLoS One. 2019 Feb 6;14(2):e0210031. doi: 10.1371/journal.pone.0210031 (PMC6364887; doi:10.1371/journal.pone.0210031)
Supplement: S3 Table — (PDF) [file pone.0210031.s003.pdf]

Table S3. Model selection results of all models depicting avian response to salmon and habitat features on 14 streams along the central coast of British Columbia.

Avian Response	Model	*K*	*logLik*	AICc	ΔAICc	*w*
All birds	Salmon, Conifer PC1	7	-304.6	624.1	0	0.31
	Salmon, Watershed, Conifer PC1	8	-303.6	624.2	0.2	0.28
	Salmon, Watershed	7	-305.0	624.8	0.8	0.21
	Salmon	6	-306.4	625.4	1.3	0.16
	Watershed, Conifer PC1	7	-307.3	629.5	5.5	0.02
	Watershed	6	-309.0	630.7	6.6	0.01
	Conifer PC1	6	-309.6	631.8	7.7	0.01
Generalists	Salmon, Shrub	7	-208.9	432.7	0	0.35
	Salmon	6	-210.5	433.7	1.0	0.21
	Salmon, Shrub, Watershed	8	-208.4	434.0	1.3	0.19
	Salmon, Watershed	7	-209.9	434.7	2.1	0.13
	Watershed, Shrub	7	-210.8	436.4	3.7	0.06
	Shrub	6	-212.2	437.1	4.4	0.04
	Watershed	6	-212.5	437.7	5.0	0.03
Insectivores	Salmon, Watershed, Red Alder	8	-290.1	597.4	0	0.34
	Salmon, Red Alder	7	-291.4	597.7	0.3	0.29
	Salmon, Watershed	7	-291.8	598.4	1.1	0.20
	Salmon	6	-293.3	599.1	1.8	0.14
	Watershed, Red Alder	7	-293.9	602.7	5.4	0.02
	Watershed	6	-295.9	604.4	7.0	0.01
	Red Alder	6	-296.5	605.6	8.3	0.01
Frugivores	Salmon, Shrub	7	-201.0	416.9	0	0.35
	Salmon	6	-202.7	418.0	1.1	0.21
	Salmon, Watershed, Shrub	8	-200.6	418.3	1.3	0.18
	Salmon, Watershed	7	-202.1	419.0	2.1	0.12
	Watershed, Shrub	7	-202.7	420.2	3.3	0.07
	Shrub	6	-204.1	420.8	3.9	0.05
	Watershed	6	-204.5	421.5	4.6	0.04
Pacific Wren	Salmon	6	-136.6	285.7	0	0.47
	Salmon, Conifer PC1	7	-136.1	287.0	1.2	0.25
	Salmon, Watershed	7	-136.5	287.8	2.1	0.17
	Salmon, Watershed, Conifer PC1	8	-136.0	289.2	3.5	0.08
	Conifer PC1	6	-140.2	293.0	7.3	0.01
	Watershed	6	-140.2	293.1	7.3	0.01
	Watershed, Conifer PC1	7	-139.6	294.0	8.3	0.01

Townsend's Warbler	Salmon	6	-98.0	208.7	0	0.39
	Salmon, Conifer PC1	7	-97.0	208.9	0.3	0.34
	Salmon, Watershed, Conifer PC1	7	-98.0	210.9	2.2	0.13
	Salmon, Watershed, Conifer PC1	8	-97.0	211.1	2.4	0.12
	Conifer PC1	6	-100.9	214.4	5.8	0.02
	Watershed, Conifer PC1	7	-100.7	216.2	7.5	0.01
	Watershed	6	-102.0	216.6	8.0	0.01
Pacific-slope Flycatcher	Watershed	6	-117.6	247.8	0	0.31
	Watershed, Conifer PC2	7	-117.1	249.1	1.3	0.16
	Salmon	6	-118.3	249.2	1.4	0.15
	Conifer PC2	6	-118.4	249.4	1.7	0.13
	Salmon, Watershed	7	-117.4	249.7	1.9	0.12
	Salmon, Conifer PC2	7	-117.9	250.6	2.8	0.08
	Salmon, Watershed, Conifer PC2	8	-117.0	251.1	3.4	0.06
Golden-crowned Kinglet	Salmon	6	-87.0	186.6	0	0.40
	Salmon, Conifer PC1	7	-86.4	187.6	1.0	0.25
	Salmon, Watershed	7	-86.5	187.9	1.3	0.21
	Salmon, Watershed, Conifer PC1	8	-86.0	189.2	2.6	0.11
	Watershed	6	-90.0	192.7	6.1	0.02
	Watershed, Conifer PC1	7	-89.6	194.0	7.4	0.01
	Conifer PC1	6	-90.9	194.5	7.9	0.01
Swainson's Thrush	Salmon, Red Alder	7	-74.1	163.1	0	0.33
	Salmon	6	-75.8	164.2	1.1	0.19
	Salmon, Watershed, Red Alder	8	-73.6	164.3	1.2	0.18
	Salmon, Watershed	7	-75.2	165.3	2.2	0.11
	Watershed, Red Alder	7	-75.5	165.9	2.9	0.08
	Red Alder	6	-77.0	166.6	3.6	0.06
	Watershed	6	-77.2	167.1	4.0	0.05
Varied Thrush	Salmon	6	-103.6	219.9	0	0.24
	Salmon, Conifer PC2	7	-102.7	220.2	0.3	0.21
	Salmon, Watershed	7	-103.1	221.0	1.1	0.14
	Salmon, Watershed, Conifer PC2	8	-102.0	221.2	1.3	0.12
	Watershed	6	-104.4	221.4	1.6	0.11
	Watershed, Conifer PC2	7	-103.3	221.5	1.6	0.11
	Conifer PC	6	-104.8	222.2	2.4	0.07
Effective Number of Species	Salmon, Conifer PC1	7	-192.4	399.6	0	0.39
	Salmon, Watershed, Conifer PC1	8	-191.6	400.2	0.6	0.29
	Salmon, Watershed	7	-193.5	401.8	2.2	0.13
	Salmon	6	-194.6	401.8	2.2	0.13
	Watershed, Conifer PC1	7	-194.9	404.6	5.0	0.03

	Conifer PC1	6	-196.9	406.4	6.8	0.01
	Watershed	6	-197.0	406.5	6.9	0.01
Richness	Salmon, Conifer PC1	7	-211.4	437.7	0	0.36
	Salmon, Watershed, Conifer PC1	8	-210.4	438.0	0.3	0.32
	Salmon, Watershed	7	-212.4	439.7	2.0	0.14
	Salmon	6	-213.7	440.1	2.4	0.11
	Watershed, Conifer PC1	7	-213.6	442.0	4.3	0.04
	Watershed	6	-215.7	444.1	6.4	0.02
	Conifer PC1	6	-215.7	444.1	6.4	0.02
