# Supplementary material for: Salmon increase forest bird abundance and diversity
Source: PLoS One. 2019 Feb 6;14(2):e0210031. doi: 10.1371/journal.pone.0210031 (PMC6364887; doi:10.1371/journal.pone.0210031)
Supplement: S4 Table — (PDF) [file pone.0210031.s004.pdf]

Table S4. Species, their associated foraging guilds, and migratory status of birds detected on point-count surveys in 2012 and 2013 along the central coast of British Columbia.

Common Name	Scientific Name	Guild	Status
Red-throated Loon*	*Gavia stellata*		
Pacific Loon*	*Gavia pacifica*		
Common Loon*	*Gavia immer*		
Great Blue Heron*	*Ardea herodias*		
Canada Goose*	*Branta canadensis*		
Common Merganser*	*Mergus merganser*		
Bald Eagle*	*Haliaeetus leucocephalus*		
Red-tailed Hawk*	*Buteo jamaicensis*		
Sooty Grouse	*Dendragapus fuliginosus*	GN	R
Sandhill Crane*	*Grus canadensis*		
Greater Yellowlegs*	*Tringa melanoleuca*		
Spotted Sandpiper*	*Actitis macularius*		
Mew Gull*	*Larus canus*		
California Gull*	*Larus californicus*		
Glaucous-winged Gull*	*Larus glaucescens*		
Marbled Murrelet*	*Brachyramphus marmoratus*		
Belted Kingfisher*	*Megaceryle alcyon*		
Rufous Hummingbird	*Selasphorus rufus*	NG	R
Red-breasted Sapsucker	*Sphyrapicus ruber*	IN	PM
Hairy Woodpecker	*Picoides villosus*	IN	R
Northern Flicker	*Colaptes auratus*	FR, GN, IN	R
Hammond's Flycatcher	*Empidonax hammondii*	IN	M
Pacific-slope Flycatcher	*Empidonax difficilus*	IN	M
Warbling Vireo	*Vireo gilvus*	IN	M
Steller's Jay	*Cyanocitta stelleri*	FR, GN, IN	R
Northwestern Crow	*Corvus caurinus*	NG	R
Common Raven	*Corvus corax*	NG	R
Tree Swallow*	*Tachycineta bicolor*		
Chestnut-backed Chickadee	*Poecile rufescens*	FR, GN, IN	R
Red-breasted Nuthatch	*Sitta canadensis*	IN	R
Brown Creeper	*Certhia americana*	IN	R
Pacific Wren	*Troglodytes pacificus*	IN	R
American Dipper*	*Cinclus mexicanus*		
Golden-crowned Kinglet	*Regulas satrapa*	IN	R

Ruby-crowned Kinglet	*Regulus calendula*	IN	M
Swainson's Thrush	*Chatharus usulatus*	FR, GN, IN	M
Hermit Thrush	*Chatharus guttatus*	FR, GN, IN	M
American Robin	*Turdus migratorius*	FR, GN, IN	R
Varied Thrush	*Ixoreus naevius*	FR, GN, IN	R
American Pipit*	*Anthus rubescens*		
Cedar Waxwing	*Bombycilla cedrorum*	FR	M
Orange-crowned Warbler	*Vermivora celata*	IN	M
Yellow Warbler	*Dendroica petechia*	IN	M
Yellow-rumped Warbler	*Dendroica coronata*	IN	M
Townsend's Warbler	*Dendroica townsendi*	IN	M
MacGillivray's Warbler	*Oporornis tolmiei*	IN	M
Common Yellowthroat	*Geothlypis trichas*	IN	M
Wilson's Warbler	*Wilsonia pusilla*	IN	M
Western Tanager*	*Piranga ludoviciana*		
Fox Sparrow	*Passerella iliaca*	FR, GN, IN	M
Song Sparrow	*Melospiza melodia*	FR, GN, IN	R
Golden-crowned Sparrow*	*Zonotrichia atricapilla*		
Dark-eyed Junco	*Junco hyemalis*	FR, GN, IN	R
Red Crossbill	*Loxia curvirostra*	GN	R
Pine Siskin	*Carduelis pinus*	FR	R

Abbreviations: GN: generalists, IN: insectivores, FR: frugivores, NG: non-guild, M: migrants, R: residents, PM: partial migrants. *Species not included in analyses because they are early spring migrants, non-forest dwelling birds, or detected on less than two surveys.
