# Supplementary material for: Salmon increase forest bird abundance and diversity
Source: PLoS One. 2019 Feb 6;14(2):e0210031. doi: 10.1371/journal.pone.0210031 (PMC6364887; doi:10.1371/journal.pone.0210031)
Supplement: S5 Table — (PDF) [file pone.0210031.s005.pdf]

Table S5. Fixed-effect estimates (standardized regression coefficients) and standard errors for averaged candidate models (ΔAIC<2) describing relative bird abundance as a function of salmon biomass, forest habitat, and watershed size along the Central Coast of British Columbia.

Avian Response	Parameter	Estimate	SE
All Birds	Salmon	0.494	0.166
	Conifer PC1	-0.127	0.073
	Year	0.093	0.057
	Watershed	0.227	0.162
Generalists	Salmon	0.404	0.166
	Shrub	0.130	0.075
	Year	-0.031	0.065
	Watershed	0.147	0.170
Insectivores	Salmon	0.529	0.179
	Watershed	0.258	0.171
	Red Alder	-0.122	0.066
	Year	0.067	0.055
Frugivores	Salmon	0.396	0.171
	Shrub	0.126	0.073
	Year	-0.164	0.063
	Watershed	0.150	0.175
Pacific Wren	Salmon	0.373	0.121
	Year	0.258	0.072
	Conifer PC1	-0.084	0.088
Townsend's Warbler	Salmon	0.399	0.134
	Year	0.025	0.073
	Conifer PC1	-0.120	0.089
Pacific-slope Flycatcher	Watershed	0.233	0.162
	Year	-0.080	0.074
	Conifer PC2	0.084	0.089
	Salmon	0.131	0.174
Golden-crowned Kinglet	Salmon	0.444	0.148
	Year	0.330	0.061
	Conifer PC1	-0.087	0.077
	Watershed	0.130	0.156
Swainson's Thrush	Salmon	0.408	0.185
	Red Alder	-0.138	0.076

	Year	-0.110	0.064
	Watershed	0.176	0.194
Varied Thrush	Salmon	0.279	0.159
	Year	0.067	0.069
	Conifer PC2	0.127	0.092
	Watershed	0.199	0.167
Effective Number of Species	Salmon	0.486	0.174
	Conifer PC1	-0.146	0.073
	Year	0.081	0.049
	Watershed	0.200	0.174
Richness	Salmon	0.465	0.176
	Conifer PC1	-0.148	0.073
	Year	0.081	0.050
	Watershed	0.233	0.176
